# APPLYING MIXED-METHODS APPROACHES TO THE STUDY OF SOCIAL TIES IN LATER LIFE

**DOI:** 10.1093/geroni/igz038.1529

**Published:** 2019-11-08

**Authors:** J Jill Suitor, Megan Gilligan

**Affiliations:** 1 Purdue University, West Lafayette, Indiana, United States; 2 Iowa State, Ames, Iowa, United States

## Abstract

This symposium brings together a diverse set of studies applying mixed-methods approaches, with an emphasis on illustrating the ways in which such designs can provide greater understanding of interpersonal processes in the middle and later years. The papers span a wide range of relational contexts, including ties between parents and adult children, grandparents and grandchildren, and couples in gay, lesbian, heterosexual partnerships. They illustrate a variety of ways to combine quantitative and qualitative data collecting using surveys, in-depth interviews, timeline data, and technological devices. In the first paper, Silverstein and Bengtson present a study of continuity, change, and conflict across the generations regarding religion. The next two papers explore the impact of social relations on well-being. Fingerman and colleagues report findings from a study of social engagement and sedentary activities; and Suitor and colleagues investigate gender differences in the effects of mothers’ favoritism on adult children’s depressive symptoms. The final two papers focus on couples’ experiences when facing potentially demanding and/or challenging life circumstances. Thomeer and colleagues present findings from a study of same-sex couples in the context of marriage and parenthood; and Umberson and colleagues shed light on marital dynamics in same-sex and different-sex couples when one partner experiences psychological distress. This diverse set of studies provides a rich overview of the ways in which mixed-methods approaches can shed more light on patterns and consequences of relationships in adulthood than could be learned using single-method designs.

